# Predicting visceral pleural invasion in lung adenocarcinoma presenting as part‐solid density utilizing a nomogram model combined with radiomics and clinical features

**DOI:** 10.1111/1759-7714.15151

**Published:** 2023-11-28

**Authors:** Fen Wang, Xianglong Pan, Teng Zhang, Yan Zhong, Chenglong Wang, Hai Li, Jun Wang, Lili Guo, Mei Yuan

**Affiliations:** ^1^ Department of Medical Imaging The Affiliated Huai'an No.1 People's Hospital of Nanjing Medical University Huai'an China; ^2^ Department of Thoracic Surgery The First Affiliated Hospital of Nanjing Medical University Nanjing China; ^3^ Department of Radiology The First Affiliated Hospital of Nanjing Medical University Nanjing China; ^4^ Shanghai Key Laboratory of Magnetic Resonance East China Normal University Shanghai China; ^5^ Department of Pathology The First Affiliated Hospital of Nanjing Medical University Nanjing China

**Keywords:** lung adenocarcinoma, nomogram, part‐solid, radiomics, visceral pleural invasion

## Abstract

**Background:**

To develop and validate a preoperative nomogram model combining the radiomics signature and clinical features for preoperative prediction of visceral pleural invasion (VPI) in lung nodules presenting as part‐solid density.

**Methods:**

We retrospectively reviewed 156 patients with pathologically confirmed invasive lung adenocarcinomas after surgery from January 2016 to August 2019. The patients were split into training and validation sets by a ratio of 7:3. The radiomic features were extracted with the aid of FeAture Explorer Pro (FAE). A CT‐based radiomics model was constructed to predict the presence of VPI and internally validated. Multivariable regression analysis was conducted to construct a nomogram model, and the performance of the models were evaluated with the area under the receiver operating characteristic curve (AUC) and compared with each other.

**Results:**

The enrolled patients were split into training (*n* = 109) and validation sets (*n* = 47). A total of 806 features were extracted and the selected 10 optimal features were used in the construction of the radiomics model among the 707 stable features. The AUC of the nomogram model was 0.888 (95% CI: 0.762–0.961), which was superior to the clinical model (0.787, 95% CI: 0.643–0.893; *p* = 0.049) and comparable to the radiomics model (0.879, 95% CI: 0.751–0.965; *p* > 0.05). The nomogram model achieved a sensitivity of 90.5% and a specificity of 76.9% in the validation dataset.

**Conclusions:**

The nomogram model could be considered as a noninvasive method to predict VPI with either highly sensitive or highly specific diagnoses depending on clinical needs.

## Graphical abstract

The study involved the steps of clinical model, radiomics model and the nomogram model construction and validation to predict part‐solid density invasive lung adenocarcinoma with visceral pleural invasion. The nomogram model could be considered as a noninvasive method to predict visceral pleural invasion (VPI) compared with other two models.

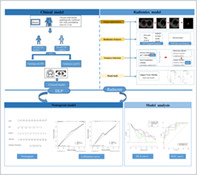

## INTRODUCTION

Visceral pleural invasion (VPI) has been regarded as an adverse prognostic factor in lung adenocarcinoma because of the potential increased risk of cancer recurrence and reduced overall survival, even in small lung neoplasms no more than 2 cm in diameter or with ground‐glass opacity.^1^, ^2^, ^3^, ^4^ The significance of VPI has been proposed in the seventh edition of the tumor‐node‐metastasis (TMN) staging system and maintained in the eighth edition of that upgrading the stage of T1 size tumor (total tumor size ≤3 cm) to affect the treatment.^5^ Yang et al.^6^ concluded that worse prognosis is associated with VPI in N0 status but disappeared in lung cancer with increasing tumor size and nodal stage. Hsu et al.^7^ showed that in making an early diagnosis of VPI, the presence of type 2 pleural tags featuring one or more linear pleural tags, with a soft tissue component at the pleural end, can reach an accuracy of 71% compared to the other two types of pleural tags. Radiological‐pathological correlation remains challenging because cancers which present on computed tomography (CT) that are in close contact with the pleura or with pleural tags do not determine the T stage.^8^ According to an observational study (JCOG0201) conducted by the Japan Clinical Oncology Group (JCOG),^9^ the presence of a ground‐glass opacity was predominantly associated with an excellent prognosis. The lung nodules whose consolidation‐to‐tumor ratio (CTR) is 0.50 or less may be deemed a radiologically noninvasive lung cancer. Because the surgical scheme of lung cancer, to some extent, depends upon the CTR, the JCOG082 study^10^ proposed that disease‐free survival resulting from sublobar resection (wedge resection or segmentectomy) for peripheral NSCLC (stage IA, CTR >0.5) is equivalent to that achieved by lobectomy. In their study, Zhao et al.^11^ concluded that VPI was more common in part‐solid than pure ground‐glass nodules (32.2% vs. 17.4%). Furthermore, Okada et al.^12^ found prognostic impact differences between pure‐ and part‐solid NSCLC; however, with only a handful of part‐solid positive cases. In previous studies^2^, ^13^ it has been reported that in pure ground‐glass opacity (pGGO) lesions, VPI was infrequently, or not, observed because of its minimally invasive quiddity of being unable to penetrate the thick elastic layer. These studies have provided useful information and inspired us to investigate whether sublobar resection may be followed by postoperative adjuvant chemotherapy or transferred to lobectomy due to the uncertain existence of VPI and lymph‐node metastasis in part‐solid lung nodules. Therefore, on the premise that the VPI of lung nodules in part‐solid lung nodules has probably affected the success rate of sublobar resection for NSCLC, preoperative VPI prediction is of vital importance.

The studies by Yuan et al.^14^ and Zha et al.^15^ attempted to derive automated quantitative imaging features to reach a noninvasive preoperative diagnosis for lung cancer with VPI hybridizing pure‐solid and solid lung lesions. Few studies have investigated radiomic signatures in only part‐solid lung adenocarcinoma. In the present study, we developed a nomogram model combined with radiomics and clinical features and internally validated for the detection of the presence of the VPI in part‐solid lung nodules with consolidation sizes of ≤3 cm.

## METHODS

### Patients and clinicopathological characteristics

This retrospective study was approved by the Ethics Committee (no.: 2021‐SRFA‐394). The requirement for written informed consent was waived because of the retrospective nature of study. The institution's medical records database was reviewed to derive the eligible patients histologically proven to have part‐solid density invasive lung adenocarcinoma with consolidation sizes of ≤3 cm (*n* = 592). Patients who received chemotherapy and radiotherapy before surgery (*n* = 0), unsatisfactory imaging quality due to respiratory artifact during examination (*n* = 3), CT examination exceeding 3‐months' time interval (*n* = 228), without preoperative CT scan (*n* = 195), or whose tumors involving other structures invaded or lymph node metastasis (*n* = 10) were excluded. A total of 156 patients with surgically resected NSCLC from January 2016 to August 2019, with consolidation sizes of ≤3 cm, were split into training and validation datasets. Among them, those patients with resected lung lesions who presented with part‐solid nodules on chest CT and had been histologically proven to be VPI positive were further selected (*n* = 70).

### Imaging protocol

All eligible patients undergoing preoperative unenhanced thin‐section CT with 1.0–1.5 mm collimation were reviewed. All CT images were performed in the supine position during inspiratory breath‐hold. The imaging parameters for thin‐section CT from different multidetector devices (Brilliance iCT [Philips Healthcare], Somatom Definition [Siemens Healthineers], GE revolution or Discovery CT [GE Healthcare]) were as follows: tube voltage 100–120 kV, automatic tube current modulation, matrix 512 × 512, slice thickness of 1–1.5 mm, the iterative reconstruction algorithm. Lung window setting of CT covers the window level of −500 to −700 HU) and a window width of the lung window (1000–1200 HU).

### Evaluation of VPI

All formalin‐fixed, paraffin‐embedded tumor specimens were implemented with routine hematoxylin and eosin staining. The surgically specimens were all invasive lung adenocarcinomas. Elastic van Gieson staining was performed to assess the presence of the visceral pleura in the cases where VPI was histologically undetermined and were re‐evaluated by a specialized pathologist (HL). According to the study by Yang et al.,^6^ there was no prognostic difference between PL1 and PL2, and therefore patients were divided into two groups based on the pathologically confirmed presence of VPI. We classified the pathological PL0 as without‐(VPI‐negative), whereas pathological PL1 and PL2 as with‐(VPI‐positive) was classified according to the criteria of VPI elucidated in a previous study.^16^

### Tumor segmentation and radiomic feature extraction

**FIGURE 1:**
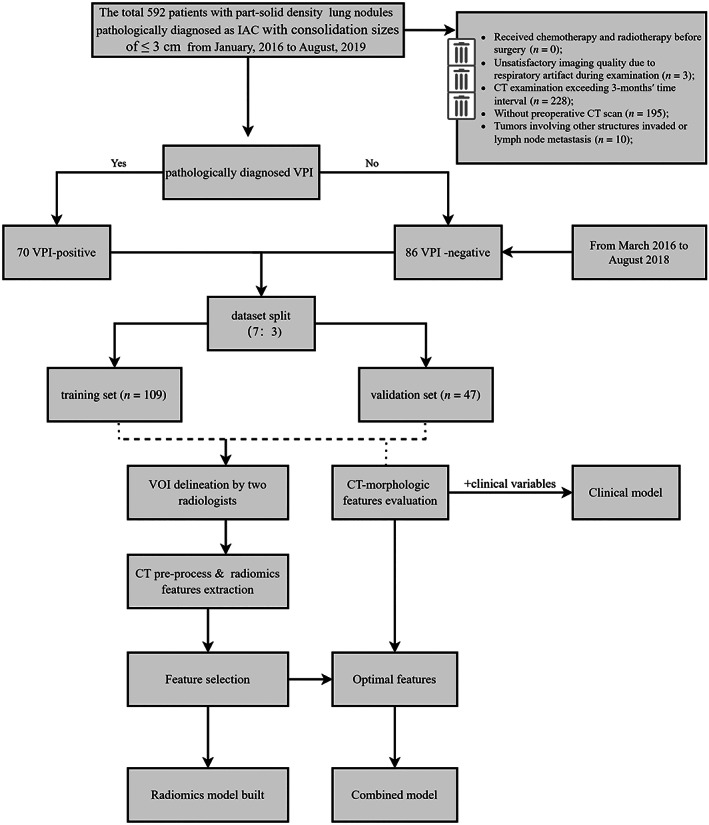
Workflow of our study. The steps involved in our study were enrolled patient selection, dataset split, radiomics model establishment, clinical model construction and combined model development.

All CT images of eligible patients were exported from the picture archiving and communication system (PACS) for image feature extraction. The volumes of interest (VOI) delineation were anonymized and semi‐automatically annotated and adjusted in exported images by a radiologist (FW) with 3 years of experience using in‐house software (multilabel; ECNU, Shanghai, China), including two steps of locating the lesion and annotating with a cooperation of semi‐automatic segmentation thresholding algorithm and a manual adjustment approach of delineation on every section of the CT scans. The radiologist repeatedly analyzed the images of 30 randomly selected patients (positive vs. negative, 1:2) twice in a 1‐month period in the same in‐house software for intraobserver reproducibility. Additionally, another radiologist (TZ) with 8 years of experience segmented 30 randomly chosen images in a blind method for interobserver reproducibility. Subsequently, the inter‐ and intraobserver reproducibility of extracted features was calculated by intraclass correlation coefficients (ICCs). The radiomic features extraction were operated from the VOIs by FeAture Explorer Pro (FAE Pro, version 0. 3.7) on Python (3.7.6).^17^ The workflow of the study is summarized in Figure 1.

### Radiomic feature‐based model construction and validation

In total, 806 high‐dimensional features were extracted. Their categories are also shown in Appendix S1. The ICCs of features greater than 0.80 were considered of good consistency and used for subsequent analysis. The radiomics model construction was based on selected features from the training cohort adopting the FAE Pro, version 0. 3.7 on Python (3.7.6). The concrete explanation of radiomic feature analysis is in Appendix S2. A radiomics score (Radscore) was computed for each patient through the value of selected features after normalization weighted by their respective coefficients.

### Clinical and nomogram model construction

All the thin‐section CT images were interpreted by thoracic radiologists (FW) who were blinded to the study design. Marginal characteristics included lobulation and spiculation signs. Internal characteristics included foam‐like and air bronchogram signs.^18^ An air bronchogram sign was classified as natural, dilated/distorted or cutoff. The pleural tag and vascular convergence signs belong to adjacent manifestation.^13^ The maximum diameter, solid component of maximum diameter, and distance from the lung nodules to the adjacent pleura (DLP) was defined as the short axial distance from the lesion to the pleura, and the longest pleural contact was also measured.^2^ Part‐solid nodules were defined as nodules containing solid and ground‐glass opacity components that were partially or completely rendered invisible when viewed with soft‐tissue window settings.^2^

We analyzed clinical or CT‐morphological features associated with VPI which were confirmed by the statistical test as the independent clinical predictors and built the clinical model by logistic regression based on the training cohort. Radiomic features were added into the optimal radiological variables to establish the nomogram model based on the multivariate logistic regression, with *p* < 0.05 associated with a backward stepwise section. A nomogram model in the training cohort was subsequently constructed. The receiver operating characteristic (ROC) curves were developed to evaluate the discriminatory performance of the nomogram model.

### Clinical usage

The calibration curve and Hosmer‐Lemeshow test were used to assess the goodness‐of‐fit of nomogram.^19^ Decision curve analysis was performed to assess the clinical value of nomogram. The net benefit is calculated within a threshold probability.

### Statistical analysis

Statistical analyses were conducted using IBM SPSS software version (version 25.0, http://www.ibm.com) and R software (version 4.1.2; http://www.r-project.org). The data were classified as the continuous variable (presented as mean ± standard deviation with normal distribution [Kolmogorov–Smirnov one‐sample test, *p* > 0.05]), otherwise, median with first and third quartiles: M (Q1,Q3) and categorical variables (presented as numbers and percent‐ages) and analyzed using a student's *t* test, Mann–Whitney U test and Chi‐square test, respectively. The intraclass correlation coefficient (ICC) was calculated on Python (3.7.6). The criteria of intraclass correlation coefficient value were graded as reported in a previous study.^19^ The performance of the prediction model was evaluated with a ROC curve and the corresponding area under the curve (AUC). The DeLong's test was also used to evaluate the difference of the ROC curves between various models. A *p*‐value <0.05 was considered statistically significant.

## RESULTS

### Clinical characteristics

A total of 156 patients (100 females, 56 males) who had lung adenocarcinoma were involved in this study. All had postoperatively confirmed invasive lung adenocarcinoma. Among them, 70 (44.9%) were diagnosed with VPI and 86 (55.1%) without VPI. The patient characteristics are shown in Table 1. A total of 109 patients were divided into a training set (positive vs. negative, 49 vs. 60) and 47 into a validation set (positive vs. negative, 21 vs. 26). The clinical data included age, tumor‐maximum diameter, and CTR did not fit the normal distribution. The age, distance from the pleural and longer pleural contact length, tumor‐maximum diameter, and CTR were calculated with the Mann‐Whitney test, resulting in the removal of age and tumor‐maximum diameter (*p* = 0.081, *p* = 0.115), respectively. The lobulation sign, air bronchogram sign and pleural tag sign were all compared with the Chi‐square test (*p* = 0.093, 0.005, 0.03, respectively). There was a significant difference in the variables between VPI‐positive and VPI‐negative group in the CTR, pleural tag sign, air bronchogram sign, distance from the pleural and longer pleural contact length (all *p* < 0.05). The independent factors predicting VPI were identified with a binary logistic regression analysis, whose corresponding regression equation was as follows: In (P/1‐P) = (−0.308) + (−0.301) × DLP + 2.099 × CTR. *P* was the probability of lung ADC with VPI‐positive. The lesion was expected to be VPI‐positive when the *P*‐value was not less than 0.5, whereas the other lesions were categorized as noninvasive lesions. The clinical model yielded the AUCs of 0.844 (95% confidence interval [CI]: 0.762–0.906) in the training set and 0.787 (95% CI: 0.643–0.893) in the internal validation set.

**TABLE 1:** Characteristics of study cohort.

Variables	Training set		Validation set	
	Positive/negative (49/60)	*p‐*value	Positive/negative (21/26)	*p*‐value
Age	61.0 (51.5, 64.0)/62.0 (54.2, 69.0)	0.081	63.0 (54.5, 66.5)/58.0 (53.5, 62.8)	0.265
Gender (male)	21 (42.9)/16 (26.7)	0.076	9 (42.9)/9 (34.6)	0.563
Lung‐tumor interface (clear)	49 (100)/60 (100)	‐	2 (4.3)/0 (0)	0.194
DLP (:mm)	0 (0, 0)/4.0 (0, 6.8)	<0.001	0 (0, 0.3)/3.7 (0, 10.1)	0.029
Longest pleural contact (:mm)	0 (0, 0.2)/5.4 (2.0, 14.2)	<0.001	0 (0, 4.0)/4.2 (0, 10.2)	0.049
Maximum diameter (:mm)	17.7 (14.7, 22.2)/20.5 (16.1, 24.7)	0.118	20.3 (17.8, 28.9)/17.3 (10.6, 23.5)	0.089
CTR	0.6 (0.4, 0.8)/0.4 (0.3, 0.6)	0.002	0.9 (0.5, 10.0)/0.4 (0.3, 0.5)	<0.001
Spiculation sign	6 (12.2)/4 (6.7)	0.340	7 (33.3)/0 (0)	0.002
Pleural tag sign	27 (55.1)/16 (26.7)	0.003	16 (76.2)/11 (42.3)	0.020
Lobulation sign	5 (10.2)/14 (23.3)	0.072	10 (47.6)/5 (19.2)	0.038
Vascular convergence sign	8 (16.3)/12 (20)	0.622	3 (14.3)/1 (3.8)	0.202
Foam‐like sign	5 (10.2)/7 (6.4)	0.240	1 (4.8)/3 (11.5)	0.408
Air bronchogram sign	22 (44.9)/27 (55.1)	0.005	13 (61.9)/15 (57.7)	0.770

*Note*: Data are mean ± standard deviation or median with interquartile range in parentheses and numbers of patients.

Abbreviations: CTR, consolidation‐to‐tumor ratio; DLP, the distance from the lung nodules to the adjacent pleural.

### Reproducibility analysis

The average ICCs of intra‐ and interobserver with satisfactory agreement were 0.899 and 0.908, respectively. Compared with the ICCs, there were 707 features with satisfactory consistency (ICC ≥0.80), 63 features with fair consistency (0.80> ICC ≥0.4) and 36 features with poor consistency (ICC ＜<0.4), respectively.

### Radiomics and nomogram model construction, validation, and evaluation

Eventually, 707 stable features were analyzed for model development after the steps of data normalization, dimension reduction, feature selection, and classifier. After reproducibility and redundancy analysis, 241 features were kept to choose optimal features. A total of 10 features were determined as the most predictive predictors after comparing the AUCs of different models on the validation dataset. Radscore was calculated by the selected features after normalization weighted by their coefficients. The formula for the Radscore was as follows:

$$“\text{Radscore}=\begin{array}{l} -0.00674+1.0035\times \mathrm{CT}\_\text{original}\_\text{firstorder}\_\text{Mean}\\ +\;1.35268\times \mathrm{CT}\_\text{original}\_\text{firstorder}\_\text{Median}\\ +\;1.62496\times \mathrm{CT}\_\text{original}\_\text{gldm}\_\text{LargeDependenceLowGrayLevelEmphasis}\\ +\;\left(-2.71250\right)\times \mathrm{CT}\_\text{original}\_\text{glszm}\_\text{ZoneEntropy}\\ +\;\left(-1.48147\right)\times \mathrm{CT}\_\text{original}\_\text{shape}\_\text{Sphericity}\\ +\;1.48313\times \mathrm{CT}\_\text{wavelet}-\mathrm{HHH}\_\text{glcm}\_\mathrm{Idn}\\ +\;1.36806\times \mathrm{CT}\_\text{wavelet}\\ -\;\mathrm{HLL}\_\text{firstorder}\_\text{Kurtosis}+0.59113\\ \times \mathrm{CT}\_\text{wavelet}-\mathrm{HLL}\_\text{glcm}\_\text{ClusterProminence}\\ +\;1.85504\times \mathrm{CT}\_\text{wavelet}\\ -\;\mathrm{LHH}\_\text{glszm}\_\text{SizeZoneNonUniformityNormalized}\\ +\;1.60010\times \mathrm{CT}\_\text{wavelet}-\mathrm{LLL}\_\text{glcm}\_{\mathrm{MCC}}^{”}. \end{array}$$

**FIGURE 2:**
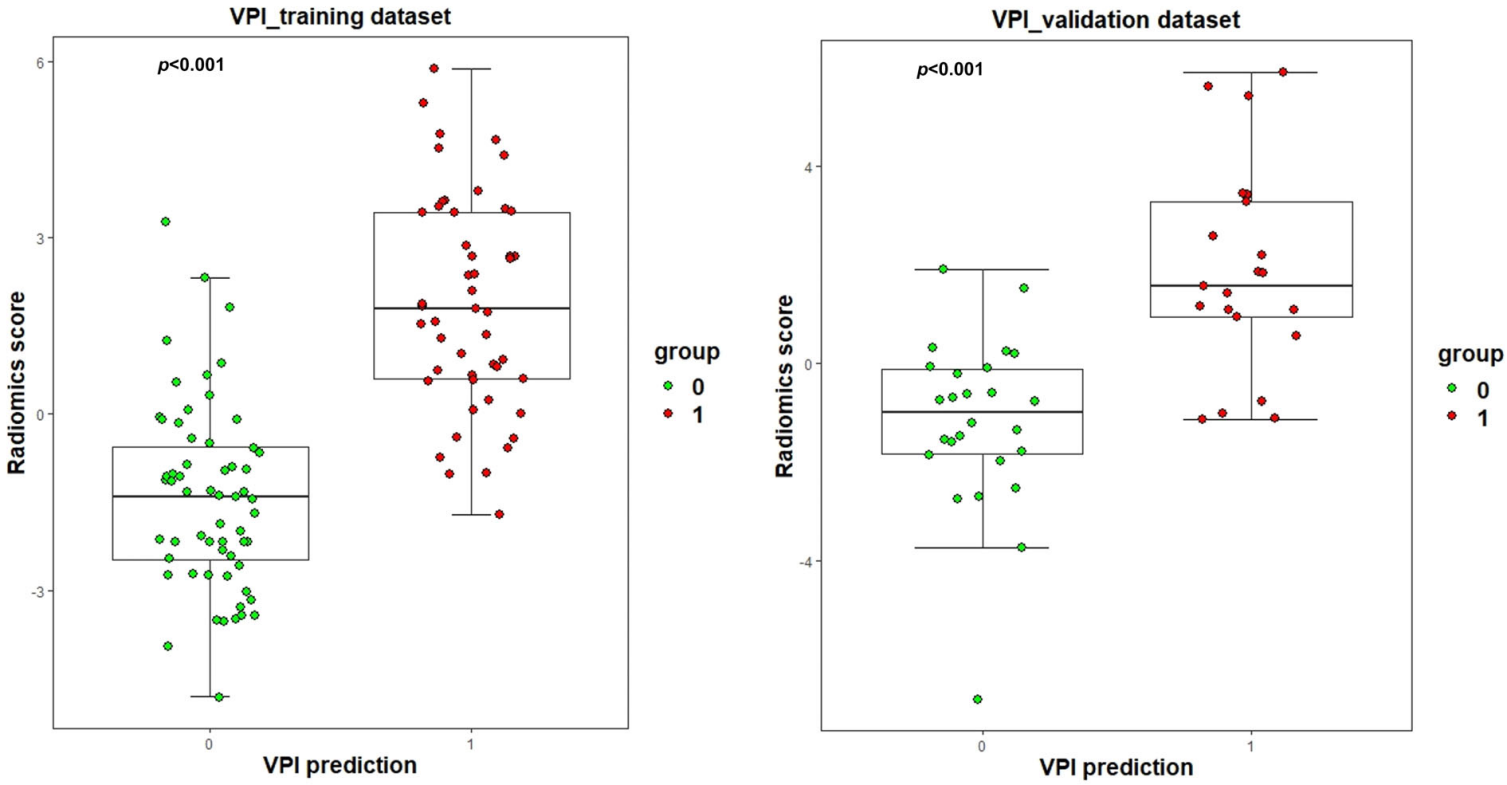
Dot gram and box plot plotted for the training and validation cohorts.

The radiomics feature model achieved an AUC (95% CI) of 0.919 (95% CI: 0.851–0.963) in the training cohort and 0.879 (95% CI: 0.751–0.956) in the internal validation cohort. The Radscore was further compared between lung adenocarcinoma with VPI and those without VPI with a significant difference in both the training and validation cohorts (Figure 2).

Then, three clinical variables (Table 1) with *p*‐values less than 0.05 combined with Radscore were used to construct a combined model with the logistic regression analysis (Table 2). The formula was: (0.721) + 1.180 × Radscore + (−0.365) × DLP.

**TABLE 2:** Logistic regression analysis for risk factors association with VPI in lung adenocarcinoma in clinical and nomogram model.

Clinical model				Nomogram model		
Variables	*β*	*p‐*value	OR (95% CI)	*β*	*p‐*value	OR (95% CI)
DLP	−0.301	<0.001	0.740 (0.627–0.873)	−0.365	0.001	0.694 (−0.561–0.858)
CTR	2.099	0.048	8.158 (1.018–65.380)	‐		
Radscore				1.180	<0.001	3.254 (1.994–5.310)

Abbreviations: CI, confidence interval; CTR, consolidation‐to‐tumor ratio; DLP, the distance from the lung nodules to the adjacent pleura; OR, overall response; Radscore, radiomics score; VPI, visceral pleural invasion.

**FIGURE 3:**
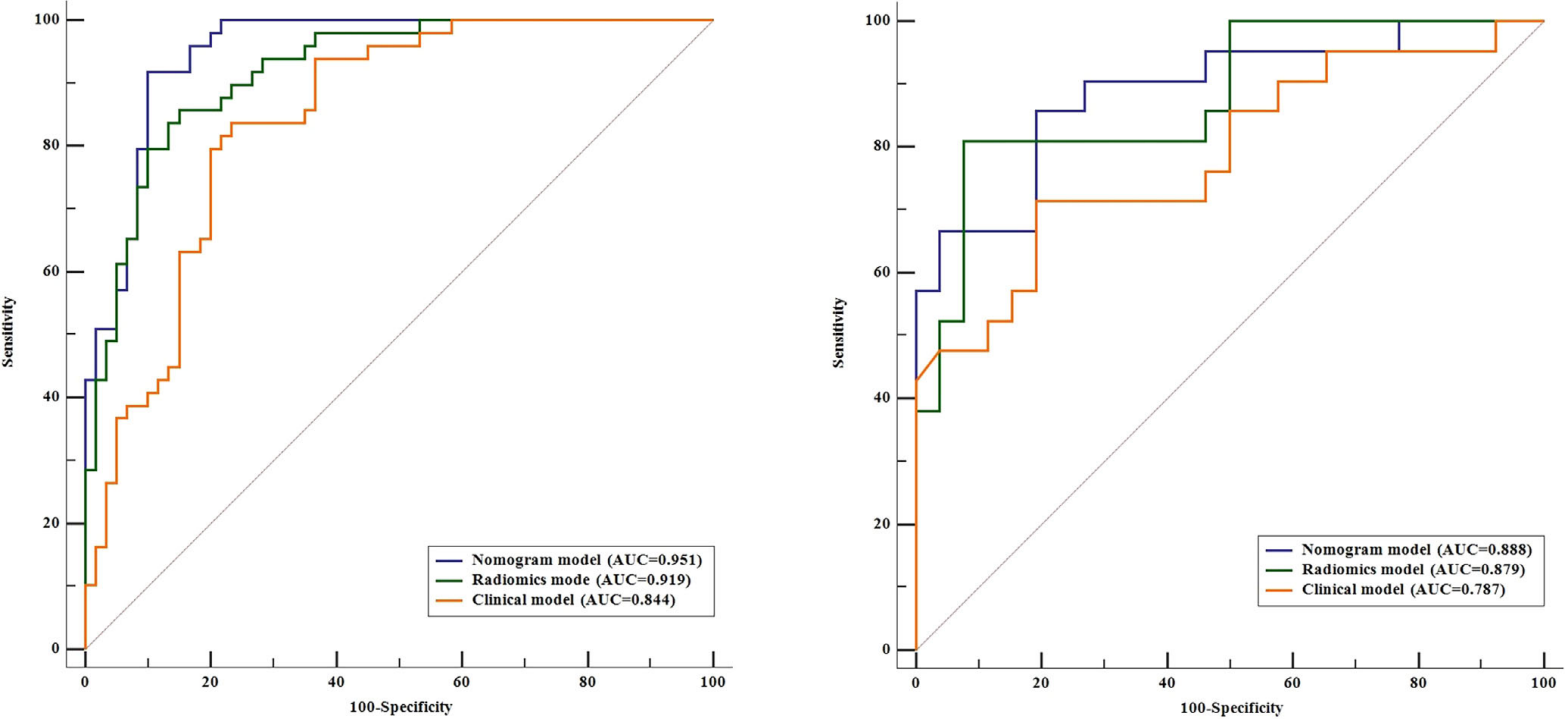
Receiver operating characteristic (ROC) curve of the established three models in two cohorts.

The AUC of the nomogram model was 0.951 (95% CI: 0.892–0.983) in the training cohort (Figure 3a) and 0.888 (95% CI: 0.762–0.961) in the validation cohort (Figure 3b). The accuracy, specificity, positive and negative predictive value, and AUCs of these models are shown in Table 3. The nomogram model outperformed the clinical feature‐based in the training dataset (*p* = 0.002) and the validation dataset (*p* = 0.049); however, it was comparable to the radiomics model in the validation dataset (*p* = 0.856). When compared with the clinical model, the radiomics model just achieved higher diagnostic efficiency, but without significant difference in the training dataset (*p* = 0.082). This trend was also observed in the validation set (*p* = 0.269) (Table 4).

**TABLE 3:** VPI‐positive diagnostic performance of three models in two cohort.

	AUC (95% CI)	SEN	SPE	ACC	PPV	NPV
Training cohort						
Nomogram model	0.951 (0.892–0.983)	83.7	76.7	79.8	74.5	85.2
Radiomics model	0.919 (0.851–0.963)	85.7	85.0	91.7	82.4	87.9
Clinical model	0.844 (0.762–0.906)	91.8	86.7	89.0	84.9	92.9
Validation cohort						
Nomogram model	0.888 (0.762–0.961)	90.5	76.9	83.0	76.0	90.9
Radiomics model	0.879 (0.751–0.956)	81.0	92.3	87.2	89.4	85.7
Clinical model	0.787 (0.643–0.893)	71.4	80.8	76.6	75.0	77.8

Abbreviations: ACC, accuracy; AUC, area under curve; CI, confidence interval; NPV, negative predictive value; PPV, positive predictive value; SEN, sensitivity; SPE, specificity; VPI, visceral pleural invasion.

**TABLE 4:** Comparison of the performance between the models.

Models (AUC)	Training set (*n* = 109)	*p‐*value	Validation set (*n* = 47)	*p*‐value
Nomogram vs. clinical	0.951 vs. 0.844	0.002	0.888 vs. 0.787	0.049
Radiomics vs. clinical	0.919 vs. 0.844	0.082	0.879 vs. 0.787	0.269
Nomogram vs. radiomics	0.951 vs. 0.919	0.039	0.888 vs. 0.879	0.856

Abbreviation: AUC, area under the curve.

### Performance of the nomogram

**FIGURE 4:**
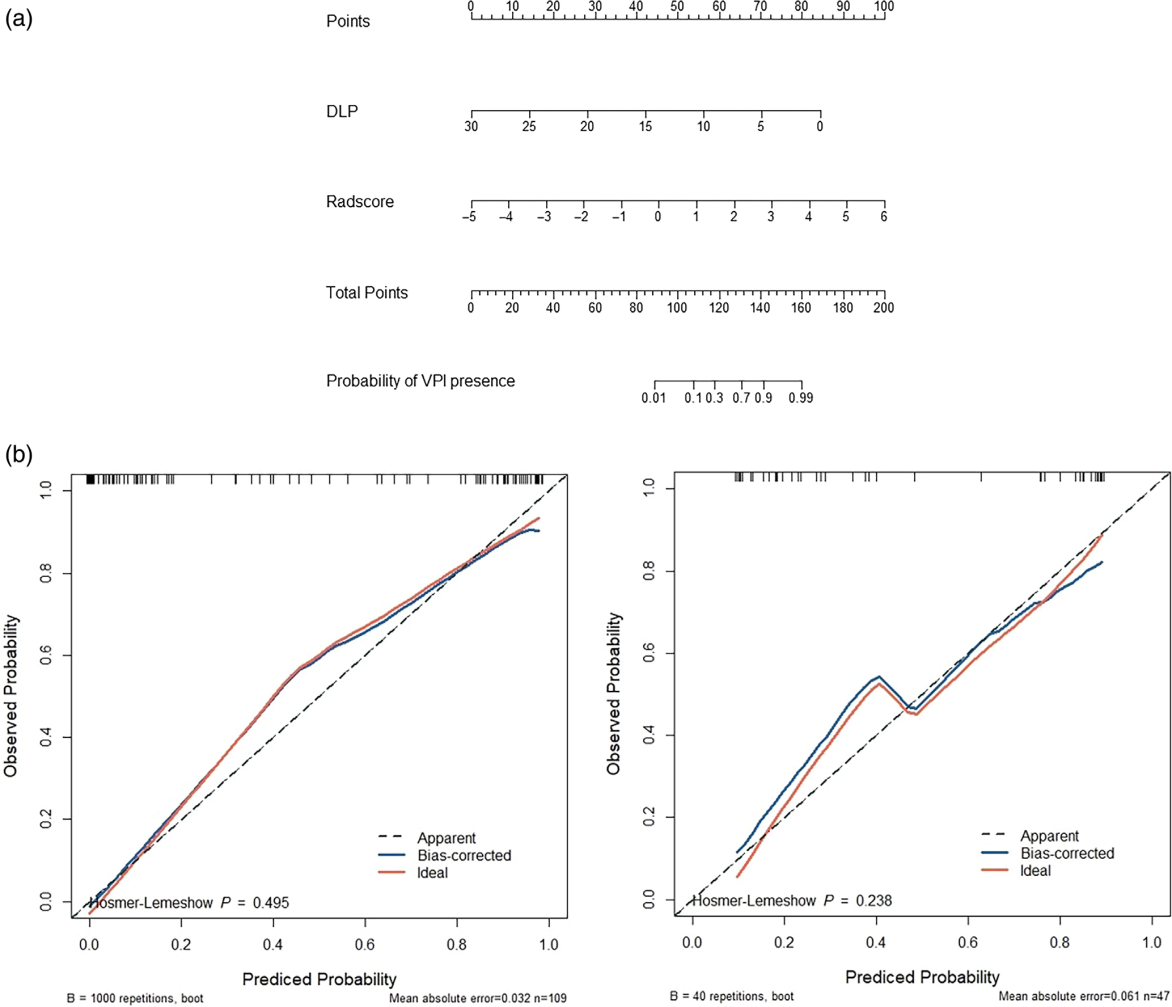
(a) The nomogram based on a combined model to predict the visceral pleural invasion (VPI)‐positive in invasive lung adenocarcinoma. (b) The calibrated curve analysis for the training and validation cohorts and the *p*‐value calculated by Hosmer‐Lemeshow test is also presented in two cohorts (*p* = 0.495 and 0.238, respectively).

**FIGURE 5:**
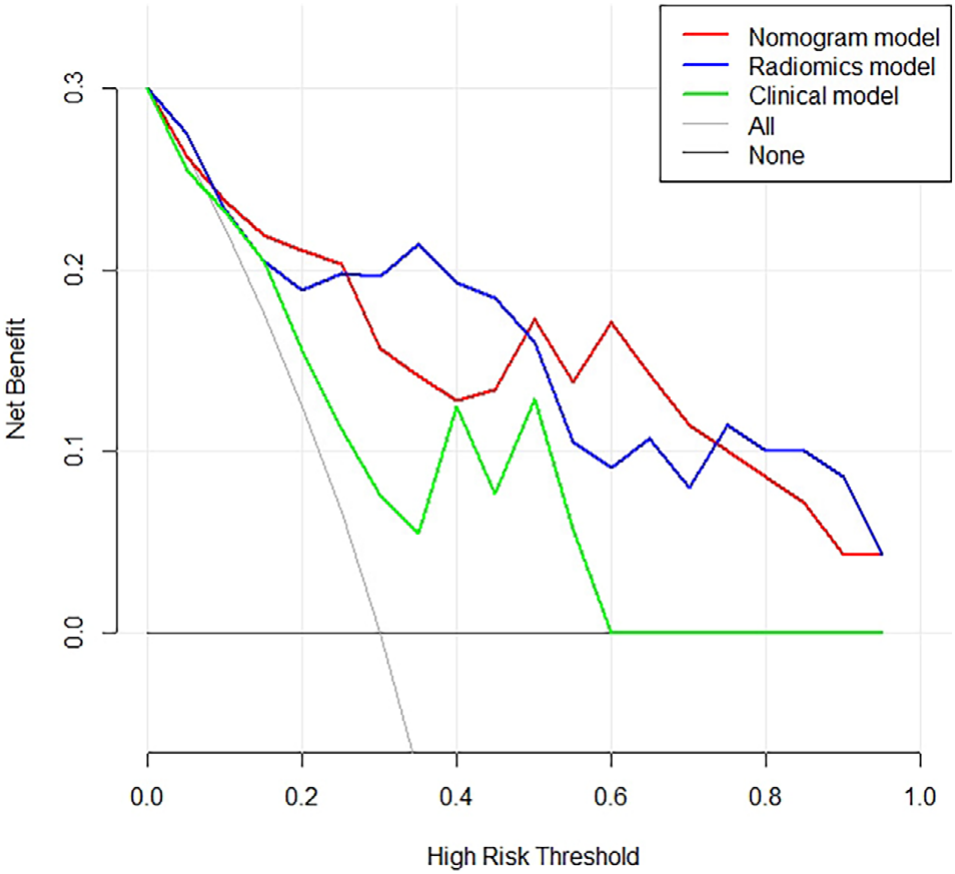
The decision curve analysis executed in the validation dataset showed that the models with Radscore provide more net benefits than the clinical model alone if the threshold probability of a patient exceeds 16%. The y‐axis indicates the net benefits and the x‐axis represents the different probability thresholds of being VPI‐positive, respectively. And the black line indicates that the net benefit is 0 and no one is treated; the gray line indicates that all samples are positive. The farther away the model curve is from these two lines, the better, and the greater the net benefit.

**FIGURE 6:**
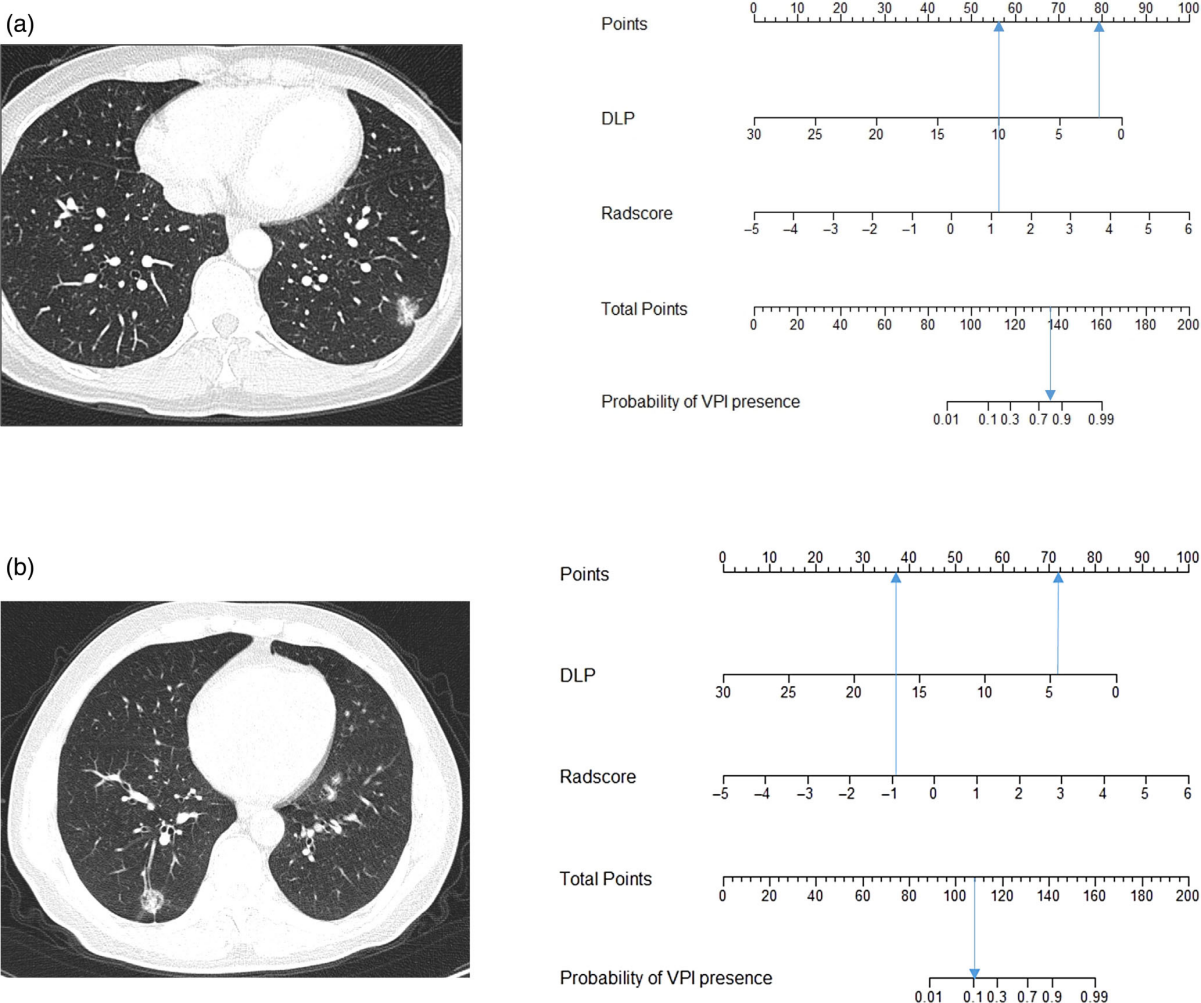
(a) A woman aged 44 with lepidic prominent invasive adenocarcinoma (IAC) in the left lower lobe with the distance from the lung nodules to the adjacent pleura (DLP) of 0.2 cm. The nomogram shows that the importance of each variable of the case was ranked according to the standard deviation along nomogram scales. To use the nomogram, the blue arrow is drawn upward to determine the points received by each variable; the sum (136) of these points is located on the total points axis, and a line is drawn downward to the probability of visceral pleural invasion (VPI) presence axes to determine the 80.23% probability of IAC with VPI‐positive. (b) A 55‐year‐old man with acinar prominent IAC in the right lower lobe with DLP of 0.43 cm. The nomogram shows that the blue arrow is drawn upward to determine the points received by each variable; the sum (109.5) of these points is located on the total points axis, and a line is drawn downward to the probability of VPI presence axes to determine the 12.40% probability of IAC without VPI.

We plotted a nomogram (Figure 4a). The calibration curve of the nomogram for predicting VPI matched well with the estimated and actual observed values of the radiomics nomogram. The *p*‐value obtained by the Hosmer‐Lemeshow test for the predictive power of the nomogram was 0.495 in the training dataset and 0.238 in the validation cohort (Figure 4b). The DCA showed that the net benefit of the combined nomogram model outperformed the clinical feature model but was comparable to the radiomics model (Figure 5). As shown in the decision curve, the combined nomogram established in this study has more benefit for predicting VPI if the threshold probability of a patient is between 8%–22% and 62%–80%. The examples of a nomogram to predict the VPI (Figure 6a,b) in invasive lung adenocarcinoma with part‐solid density are exhibited.

## DISCUSSION

Based on the discussion of a surgical scheme for part‐solid lung nodules^6^ and potential risk for VPI and lymph node metastasis in lung nodules, this retrospective study evaluated the predictive performance of a nomogram model based on clinical and radiomic features extracted from CT imaging for identifying VPI in part‐solid density lung adenocarcinoma. In this study, we constructed a nomogram model to distinguish the presence of VPI in a cohort of 156 patients with part‐solid density lung adenocarcinoma. A total of 806 quantitative radiomics signatures were extracted and processed in order to establish a radiomics model. The nomogram model was anchored on the combination of clinical radiological predictors and radiomic features, which is superior in the classification of stage I lung adenocarcinoma in those patients with VPI and without VPI, with an AUC value greater than those of the radiomics (0.879 [95% CI: 0.751–0.956]) and the clinical models (0.787 [95% CI: 0.643–0.893]). The results demonstrated that the nomogram model can reliability predict VPI with AUC (0.888 [95% CI: 0.762–0.961]) and specificity (90.5%), sensitivity (76.9%) and accuracy (83.0%). The CT‐based radiomic features, converted into Radscore, can be an independent indicator of VPI‐positive prediction in patients with lung adenocarcinoma.

The eighth edition of the American Jiont Committee on Cancer (AJCC) staging reported that a tumor size of 0–3 cm with VPI (including PL1 and PL2) is upgraded to IB stage. In the context of clinical treatment, previous studies have found that patients with stage IB NSCLC can benefit from adjuvant chemotherapy treatment. Therefore, if part‐solid lung nodules are removed by sublobar resection as suggested in the JCOG082 study, then patients with part‐solid nodules with VPI should be suitable to proceed with adjuvant chemotherapy treatment. VPI in lung adenocarcinoma possibly correlates to patient prognosis.^3^ Pathological diagnosis has been the gold standard for the examination of the presence of the VPI. As a result, clinicians have had to find a noninvasive way according to the different radiology modalities to reach a preoperative diagnosis for patients. Yang et al.^6^ proposed that the presence of VPI can be a negative prognostic factor for T1‐2N0M0 NSCLC and has an effect on cancer‐specificity survival. Kudo et al.^20^ affirmed that patients with VPI are likely to have more extensive hilar or mediastinal lymph node metastases and further hypothesized that lymphatic vessels are widespread in VPI and develop an intercommunicating network on the lung surface and are able to penetrate lung parenchyma with bronchial lymph vessel drainage to hilar lymph node.

The correlation between preoperative CT morphological features and the presence of VPI has been investigated. In the present study we built a nomogram model which revealed that the lobulation and air bronchogram signs were not predictors of VPI in lung adenocarcinoma. Our clinical model showed that indicators for assessing VPI in part‐solid tumor in lung adenocarcinoma are the DLP and CTR. Nevertheless, the DLP and CTR were not considered predictors of VPI in lung adenocarcinoma based on the maximum standard unit value of ^18^F‐fluorodeoxyglucose positron emission tomography/computed tomography and computed tomography features in the study by Wang et al.,^21^ and we attribute this phenomenon to the enrolled lung lesion including the solid and part‐solid density. The lobulation and air bronchogram signs were also not considered to be predictors for VPI evaluation in agreement with previous studies.^14^, ^22^ A lobulated sign can be a good indicator of malignancy of lung neoplasm. It should be noted that it may also possibly occur in benign nodules.^23^ The air bronchogram sign means that the tumor is spreading along the wall of the fine bronchus and alveolar wall without destroying the lung structure. Prior studies have revealed that the air bronchogram sign could be a morphological manifestation to distinguish the presence of VPI in lung adenocarcinoma.^24^ In reality, the small number of enrolled patients in this study also contributed to the selection bias. According to the study by Yang et al.,^6^ the pleural tag sign manifesting one or more bold‐wire pleural tags with soft tissue components at the pleural end and a tumor that pushes the pleura is widely associated with VPI. Current studies based on morphological features of CT images remain undetermined that lung nodules in close contact to the pleura or with pleural tags can be upstaged from T1 to T2 stage clinically.^25^ Our study internally investigated whether the pleural tag sign is a predictor for the VPI in patients with part‐solid lung carcinoma, the results of which correspond with previous studies,^2^ and it is supposed that the solid component of part‐solid lung nodule is not relatively too high to make the pleural tag sign so obvious. The CTR is the radiological manifestation of the solid component of part‐solid nodules, and our study found that the CTR was a predicted indicator for VPI mainly because the CTR can be indirectly related to the aggressiveness of part‐solid nodules and the CTR more than 0.5 (56.5% in the training set and 72.7% in the validation set), respectively of enrolled eligible nodules was dominant in the VPI‐positive group.

At present, radiomics enables large volumes of complex quantitative imaging features from CT images to be examined in order to identify precise characteristics or evaluation of internal tumor heterogeneity.^2^, ^14^, ^26^, ^27^ Yuan et al.^14^ proposed the integration of pathological findings and multifeature‐based radiomics to evaluate potential malignant characteristics and prognostic factors for discriminating VPI in NSCLC with tumors 3 cm or less in size. However, the model just devoted to a couple of prognostic parameters and did not take more morphological features into account. In this study, 806 features were extracted from all thin‐slice images. After reproducibility and redundancy analysis, 241 features were kept to subsequently select optimal features. Eventually, 10 optimal quantitative radiomic features were extracted. This study covered first‐ to high‐order texture features which is partially consistent with a previous study by Wei et al.,^28^ suggesting some similarities between the two studies regarding texture features, but not in agreement with the study by Zha et al.^15^ According to our study, the model combination with radiomic and clinical features is more effective. Due to the difficulty in delineating peritumoral ROI for the reason of the proximity of the tumor to the pleura, we only annotated the interior VOI. However, some imaging features were thought to reflect the peritumoral condition as mentioned previously,^13^ which is consistent with previous studies that the above features are the important factors of VPI. We found that the radiomics model obtained higher accuracy (91.7%, 87.2%, respectively) in the training and validation cohorts than the other two models.

The pairwise Delong's test assessment showed that the radiomics (AUC = 0.879) and the nomogram models (AUC = 0.888) had similar diagnostic efficiency; however, both AUC values of the clinical model in the training and validation sets were lower than that of the nomogram model and embodied the significant difference (*p* < 0.05). The similarity in the performance of the clinical model and radiomics model principally because that we include some peritumoral morphological signs and the radiological features are confirmed carefully by radiologist. Furthermore, the DCA curve analysis showed that the model with Radscore was superior to the model without Radscore (clinical model) within a threshold probability range above 16%. The phenomenon was also present in another study devoted to investigating the invasive prediction within lung neoplasm with the aid of radiomics signature.^29^

Our study had certain limitations. First, it was a retrospective study with potential selection bias. Second, volumetric segmentation may be not feasible or completely reproducible in the clinical setting and is still a time bottleneck. More work needs to be done to validate the robustness and ruggedness of our prediction model. Third, multiple different CT scanning devices were used employing different acquisition protocols.

In conclusion, a CT image‐based nomogram model combining radiomic features and clinical features was conducted for predicting VPI in lung adenocarcinoma internally. A nomogram model based on radiomic features may provide a noninvasive method to evaluate the prognosis of invasive lung adenocarcinoma.

## AUTHOR CONTRIBUTIONS

Fen Wang: Conceptualization, data curation, investigation, methodology, project administration, software, writing–original draft; writing–review and editing, visualization, final approval of the manuscript. Xianglong Pan: Conceptualization, data curation, visualization, writing–review and editing, final approval of the manuscript. Teng Zhang: Conceptualization, data curation, investigation, visualization, writing–review and editing, final approval of the manuscript. Chenglong Wang: Conceptualization, data curation, investigation, methodology, software, final approval of the manuscript. Yan Zhong: data curation, software, visualization, final approval of the manuscript. Hai Li: Conceptualization, final approval of the manuscript. Jun Wang: Conceptualization, data curation, investigation, project administration, supervision, visualization, writing–review and editing, final approval of the manuscript. Lili Guo: Conceptualization, data curation, investigation, project administration, supervision, final approval of the manuscript. Mei Yuan: Conceptualization, data curation, investigation, project administration, supervision, visualization, writing–review and editing, final approval of the manuscript.

## FUNDING INFORMATION

This research did not receive any specific grant from funding agencies in the public, commercial, or not‐for‐profit sectors.

## CONFLICT OF INTEREST STATEMENT

The authors declare no conflicts of interest.

## Supporting information


**Appendix S1.** The categories of elucidated extracted radiomic features.
**Appendix S2.** The concrete explanation of radiomic features analysis.
**Figure S1.** The process of VOI segmentation. (a) Mark the lesion automatically with the in‐house software. (b) Manual correction of lesions. (c) The eventually save format (NII) for the subsequent features extraction.
