# Supplementary material for: Predicting visceral pleural invasion in lung adenocarcinoma presenting as part‐solid density utilizing a nomogram model combined with radiomics and clinical features
Source: Thorac Cancer. 2023 Nov 28;15(1):23–34. doi: 10.1111/1759-7714.15151 (PMC10761615; doi:10.1111/1759-7714.15151)
Supplement: Supplementary file 1 — Appendix S1. The categories of elucidated extracted radiomic features. Appendix S2. The concrete explanation of radiomic features analysis. Figure S1. The process of VOI segmentation. (a) Mark the lesion automatically with the in‐house software. (b) Manual correction of lesions. (c) The eventually save format (NII) for the subsequent features extraction. [file TCA-15-23-s001.docx]

Supplementary Materials

**Supplementary Methods**

**Appendix 1. The categories of elucidated extracted radiomics features**

The categories of them are as follows: first order statistics (n=18), shape (n=14), grey level dependence matrix (GLDM) (n =14), grey level co-occurrence matrix (GLCM) (n =24), Gray level run-length matrix, (GLRLM) (n=16), and grey level zone size matrix (GLZSM) (n =16) and wavelet features(n=704).

**Appendix 2. The concrete explanation of radiomics features analysis.**

The feature matrix was all loaded in FAE and the dataset was split by 7:3 ratio into a training dataset 109 of samples and an independent test dataset of 47 samples. To Remove the unbalance of the training data set, we up-samples by repeating random cases to make VPI positive/negative samples balance. All images were performed image normalization procedure before feature extraction. Secondly, each feature is scaled to [0,1] using mean normalization technique. Thirdly, feature selection and dimension reduction are based on the method of Recursive Feature Elimination (RFE) and Pearson Correlation Coefficient (PCC), respectively. Finally, Support Vector Machine (SVM) was used to select the most optimal features of model-building. A 5-fold cross-validation was conducted to ensure the stability and reliability of our established model.

**Supplementary Figures**

**Figure S1**


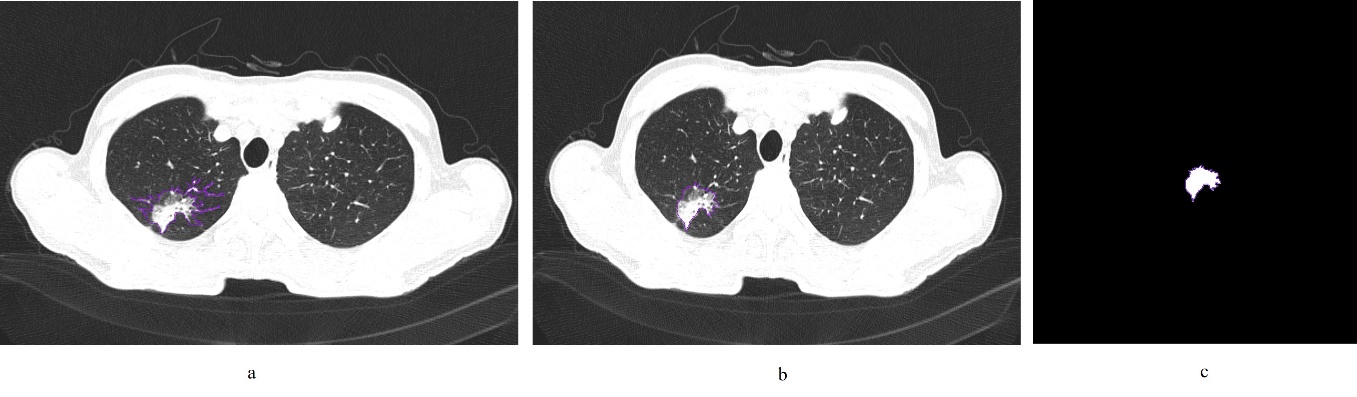
**Figure S 1.** The process of VOI segmentation. (a) Mark the lesion automatically with the in-house software. (b) Manual correction of lesions. (c) The eventually save format (NII) for the subsequent features extraction
